# Mental health care for irregular migrants in Europe: Barriers and how they are overcome

**DOI:** 10.1186/1471-2458-12-367

**Published:** 2012-05-20

**Authors:** Christa Straßmayr, Aleksandra Matanov, Stefan Priebe, Henrique Barros, Reamonn Canavan, José Manuel Díaz-Olalla, Edina Gabor, Andrea Gaddini, Tim Greacen, Petra Holcnerová, Ulrike Kluge, Marta Welbel, Pablo Nicaise, Aart H Schene, Joaquim JF Soares, Heinz Katschnig

**Affiliations:** 1 Ludwig Boltzmann Institute for Social Psychiatry, Lazarettgasse 14A-912, 1090 Vienna, Austria; 2 Unit for Social and Community Psychiatry, Queen Mary University of London, London E13, 8SP Mile End Road, London, UK; 3 Department of Clinical Epidemiology, Predictive Medicine and Public Health, University of Porto Medical School, Al Prof Hernani Monteiro, 4200-319, Porto, Portugal; 4 Health Promotion Research Centre, National University of Ireland Galway, University Road, Galway, Ireland; 5 Madrid Salud, Calle Juan Esplandiú nº 13, 28007 Madrid, Spain; 6 National Institute for Health Development, 1096 Budapest Nagyvárad tér 2, Budapest, Hungary; 7 Laziosanità ASP - Public Health Agency, Lazio Region, Via di S. Costanza 53, 00198 Rome, Italy; 8 Laboratoire de recherche, Etablissement public de santé Maison Blanche, 18 rue Rémy de Gourmont, 75019 Paris, France; 9 Department of Psychiatry, 1st Faculty of Medicine, Charles University, Ke Karlovu 11/12000, Prague, Czech Republic; 10 Clinic for Psychiatry and Psychotherapy, Charité, University Medicine Berlin, CCM, Charitéplatz 1, 10117 Berlin, Germany; 11 Institute of Psychiatry and Neurology, ul. Sobieskiego 9, 02-957 Warsaw, Poland; 12 Institute of Health and Society (IRSS), Université Catholique de Louvain, Clos Chapelle- aux-Champs, 30.05, B-1200 Bruxelles, Belgium; 13 Academic Medical Center, University of Amsterdam, Meibergdreef 5, Room PA1-156, 1105 AZ Amsterdam, The Netherlands; 14 Department of Public Health Sciences, Karolinska Institute, Norrbacka, SE-171 76 Stockholm, Sweden and Department of Public Health Sciences, Mid Sweden University, SE-851 70 Sundsvall, Sweden

**Keywords:** Irregular migrants, Mental health care, Access to care, Barriers to care, Legal entitlement, Overcoming barriers, Europe

## Abstract

**Background:**

Irregular migrants (IMs) are exposed to a wide range of risk factors for developing mental health problems. However, little is known about whether and how they receive mental health care across European countries. The aims of this study were (1) to identify barriers to mental health care for IMs, and (2) to explore ways by which these barriers are overcome in practice.

**Methods:**

Data from semi-structured interviews with 25 experts in the field of mental health care for IMs in the capital cities of 14 European countries were analysed using thematic analysis.

**Results:**

Experts reported a range of barriers to mental health care for IMs. These include the absence of legal entitlements to health care in some countries or a lack of awareness of such entitlements, administrative obstacles, a shortage of culturally sensitive care, the complexity of the social needs of IMs, and their fear of being reported and deported. These barriers can be partly overcome by networks of committed professionals and supportive services. NGOs have become important initial points of contact for IMs, providing mental health care themselves or referring IMs to other suitable services. However, these services are often confronted with the ethical dilemma of either acting according to the legislation and institutional rules or providing care for humanitarian reasons, which involves the risk of acting illegally and providing care without authorisation.

**Conclusions:**

Even in countries where access to health care is legally possible for IMs, various other barriers remain. Some of these are common to all migrants, whilst others are specific for IMs. Attempts at improving mental health care for IMs should consider barriers beyond legal entitlement, including communicating information about entitlement to mental health care professionals and patients, providing culturally sensitive care and ensuring sufficient resources.

## Background

Irregular migrants (IMs) are one of the most socially marginalised groups in Europe. Policies pertaining to this group often attract extensive public debate. In some countries, IMs are perceived and/or portrayed as a threat to society, while in others they are silently tolerated. Whilst at a European level recommendations to improve health care for migrants in general exist [1,2], the actual commitment to providing health care for IMs lags behind and varies considerably between countries. In 2011 a resolution on “Reducing health inequalities in the EU” was adopted by the European Parliament. This document specifically urges Member States to focus on health needs of IMs amongst other vulnerable groups [3].

“Irregular migration” is a form of migration in which the rules of entry or residence have been disregarded at some point during the migration process. Migrants can acquire an irregular status by birth, illegal entry, overstaying or status withdrawal [4]. The term “irregular migrants” has been introduced to avoid the discriminatory, dehumanising and criminalising term “illegal migrants”; sometimes the terms “undocumented migrants” [5] or “migrants in an irregular situation” [6] are also used.

Migration into and within European countries is a rapidly growing phenomenon, and there is an increasing literature on access to and provision of health care for migrants. For the subgroup of IMs, a number of studies on general health care have been conducted in recent years [7-14]. However, despite the evidence that being an IM is associated with high levels of psychological stress and increased risk of mental health problems [13,15,16], research on mental health care provision for this group is limited. IMs are confronted with poverty, uncertainty, social isolation, and exploitation in the illegal labour market. They are also often deprived of basic standards of living [5,14,17,18]. Castañeda [13] coined the term “illegal syndrome” for describing the strain on mental health due to living a life in illegality. Mental health was the most frequently reported health need of IMs in the 17 EU countries studied in the recently conducted “Health Care in NowHereland” project, which aimed at generating evidence on policies, practices and experiences of health care for IMs in Europe [8,19]. In this project a classification system based on the level of legal entitlements to health care in each EU country was proposed. Austria, the Czech Republic, Germany, Hungary, Ireland, Poland and Sweden were classified as “no access countries”, which provided no legal access to health care for IMs except for emergency interventions. Germany was assigned to the “no access group” due to the obligation in that country at the time of the study to report IM patients, which significantly limited actual access to care. Belgium, Italy and the United Kingdom (UK) were classified as “partial access countries”. This meant that IMs had access to some types of services beyond emergency care, for example primary care and/or services for specific groups (e.g. children, pregnant women), but not to all services. France, The Netherlands, Portugal and Spain were classified as “full access countries”, providing the same access of health care to IMs as to nationals [19].

The aim of the present paper was to explore the views of experts in mental health care for IMs on access to and provision of mental health services for this group in 14 European countries. The focus was on identifying barriers to mental health care for IMs and the ways by which these are overcome in practice.

## Methods

### Data collection

Data was collected by conducting semi-structured interviews with experts on mental health care for IMs as part of project “Best Practice In Promoting Mental Health In Socially Marginalised People In Europe” (PROMO, http://www.promostudy.org). The project was funded by the European Commission. It was carried out between 2007 and 2010 in the capitals of 14 European countries: Austria, Belgium, Czech Republic, France, Germany, Hungary, Italy, Ireland, The Netherlands, Poland, Portugal, Spain, Sweden and UK. It addressed care for six marginalized groups: long-term unemployed, homeless, asylum seekers/refugees, sex workers, travelling communities, and IMs. The aim of the PROMO study was to assess service provision for these groups in two highly deprived areas of each participating capital and also to identify components of good practice in mental health care.

### Experts

For the study on mental health care for IMs we aimed to interview one expert for each included deprived area (28 in total). Experts were selected through contacts with relevant services providing care for IMs. The inclusion criteria were: knowledge of local service provision in deprived areas of the respective capital; expertise of providing or facilitating access to mental health care for IMs; and a professional background in mental health, general health or social care. Ethical approval was not required, as no patient data was collected and expert views were anonymised.

### Semi-structured interviews

Experts were interviewed using a semi-structured, vignette-based interview schedule, which was developed in cooperation with all PROMO partners. Pilot interviews were carried out in each participating centre, before the final version was produced. The schedule was translated into the languages of all participating countries. The first part of the schedule contained two case vignettes which presented different clinical conditions, followed by open questions on the pathways to services, barriers encountered by IMs and ways to overcome them (Table 1). The experts were asked to refer to these case vignettes when answering the questions. The second part of the schedule consisted of general questions relating to the overall quality of mental health care for IMs in the given area. The experts were asked about the coordination of care at the administrative and the individual patient level, the strengths and weaknesses of care provided to IMs, and, finally, suggestions for improving the quality of care for this group.

**Table 1:** Part 1 of the questionnaire for the interviews with experts on mental health care for irregular migrants

***Vignette 1***	
A 30-year old male, an irregular migrant from Uganda, has been in the country for an unknown period of time.	
He hears voices and appears disturbed. He is socially isolated, talks using incoherent sentences, has poor personal hygiene, and has *not* tried to get in contact with services.	
1. Who would be likely to notice his problems and initiate help?	
2. Which services/organisations would, once informed, go out and contact him?	
3. What care would they provide, or how would they refer the person on?	
4. What are the further care pathways and/or treatment options for him?	
5. What are the barriers for him to receive that care and/or treatment?	
6. Are there any ways to overcome these barriers?	
a) Are there any ways for the *client* to overcome these barriers?	
b) Are there any ways for the *service* to overcome these barriers?	
***Vignette 2***	
A 40-year female, an irregular migrant from Uganda, has been in the country for an unknown period of time. She is living alone, and is depressed with suicidal ideation. She wants help.	
1. How would she find information on how to get help for her mental health problem?	
2. Which services/organisations could she approach?	
3. What are the further care pathways and/or treatment options for this person?	
4. What are the barriers for her to receive that care and/or treatment?	
5. Are there any ways to overcome these barriers?	
a) Are there any ways for the *client* to overcome these barriers?	
b) Are there any ways for the *service* to overcome these barriers?	

#### Procedure

The experts were contacted by the researchers in each participating centre via telephone or email. A detailed description of the study was provided to all potential participants, informed consent was obtained, and confidentiality and anonymity were assured. All interviews were carried out face-to-face and audio-taped by the researchers in the participating centres. The average length of interviews was 30 minutes. The occupational background of the interviewees and the nature of their professional involvement with socially marginalised groups were documented. The interviews were transcribed and translated into English by researchers in participating centres, removing any identifying information to maintain anonymity.

### Data analysis

The transcripts of the interviews were analysed using thematic analysis, a qualitative research method for the interpretation of text data through the systematic classification process of coding and identifying themes or patterns [20,21]. Analyses were carried out by four project research members in two participating countries (Austria and UK). The background of the core team analysing the data included research in public health, sociology, psychology, management of health services, and academic and clinical work in social psychiatry. Discrepancies were discussed at each stage of the analysis. In some instances additional clarification was required from the researchers of the partner countries. In the first stage of the analysis, codes were developed by reading the text of six transcripts line by line. The resulting coding frame was then used to code all the transcripts. When new codes were identified, they were discussed and incorporated into the existing coding frame. The next stage of the analysis consisted of merging codes into categories and subsequently refining them and grouping them into conceptual themes [22,23]. Frequency counts of the related themes were recorded [24]. Finally, all partner countries were asked to check the results for consistency.

## Results

We identified and assessed 25 experts in 14 European capitals: Vienna, Brussels, Prague, Paris, Berlin, Budapest, Rome, Dublin, Amsterdam, Warsaw, Lisbon, Madrid, Stockholm and London. Two experts were assessed in each city, apart from London, Stockholm and Vienna, where one expert provided assessments for both deprived areas. Twelve experts were currently employed in health care services, eleven in some type of social care service, and two experts were from research centres. The interviewed experts had on average 12 years of experience of care for IMs (median: 10 years).

The results are presented according to the two main research questions: (1) barriers to mental health care for IMs migrants in Europe and (2) ways these barriers are overcome in practice. While the themes presented are distinct, the complexity of barriers to mental health services for IMs has resulted in a certain degree of overlap.

### Barriers to mental health care

We identified eight themes related to barriers to accessing and receiving mental health care for IMs (Table 2).

a. *No legal entitlement to mental health care – emergency care only*

**Table 2:** Barriers to mental health care - frequencies of themes sorted by the number of countries from highest to lowest

**Themes**	**14 countries 25 experts**	**Countries**
IMs’ complex needs beyond the capacity of mental health services (h)	11 countries 16 experts	Austria, Belgium, France, Germany, Ireland, Italy, The Netherlands, Poland, Portugal, Spain, United Kingdom
Lack of trust in health and social care institutions (g)	11 countries 16 experts	Austria, Belgium, Czech Republic, France, Germany, Ireland, The Netherlands, Poland, Portugal, Sweden, United Kingdom
General shortage of resources in services providing mental health care (f)	10 countries 12 experts	Austria, Belgium, France, Ireland, Italy, The Netherlands, Poland, Portugal, Spain, United Kingdom
Lack of specialised resources for treating migrants with mental health problems (e)	9 countries 12 experts	Austria, Belgium, France, Hungary, Ireland, The Netherlands, Poland, Portugal, Spain
Care providers’ lack of awareness of the legal entitlement of IMs to mental health care (d)	8 countries 12 experts	Austria, Belgium, France, Germany, Ireland, The Netherlands, Portugal, United Kingdom
No legal entitlement to mental health care – emergency care only (a)	7 countries 12 experts	Austria, Czech Republic, Germany, Hungary, Ireland, Poland, Sweden
Administrative barriers obtaining mental health care even if legal entitlement exists (b)	7 countries 11 experts	Belgium, Germany, Italy, The Netherlands, Portugal, Spain, United Kingdom
IMs’ lack of awareness of entitlement to mental health care (c)	6 countries 8 experts	Belgium, Ireland, The Netherlands, Poland, Portugal, United Kingdom

Experts from the countries that provide no legal access to mental health care for IMs beyond emergency care described this lack of legal entitlement as the main barrier to mental health care for this group:

*“Illegal immigrants have no rights to anything, that they have to hide and that it is really a hard time for them. So their biggest problem is the legal situation – they do not have their documents, they feel like they have lost their dignity, they feel like they were shadows. The barriers are everywhere: inside, outside. They do not have legalaccess to public health care and free medical services. And they do not have the means to pay for it themselves.” (Poland_121)*"

In these countries provision of mental health care to IMs rarely consists of care beyond mandatory crisis intervention. Follow up appointments and continuous forms of care, which are typically essential in the treatment of patients with more severe mental illnesses, are therefore not possible. The lack of legal entitlement also means that IMs frequently delay visiting health services until their illness reach a serious stage.

b. *Administrative barriers obtaining mental health care even if legal entitlement exists*

In countries where legal access to health care for IMs is in place, obstacles in the form of administrative barriers were often reported. Some experts considered additional administrative procedures required for cost reimbursement for the treatment of IMs a burden for services. Administrative requirements and complex procedures for obtaining health insurance or registering with the given national health system were seen as limiting access to care in practice. For instance, irregular migrants may not succeed in obtaining a health card or registering with a general practitioner because they do not have all the necessary documents or a permanent address.

c. *IMs’ lack of awareness of entitlement to mental health care*

*“If he [an IM] has no fixed address then he might not already have a general practitioner, and sometimes they can put that up as a barrier to secondary services… So lack of general practitioner could be an issue.” (UK_173)*"

Experts noted that IMs often arrive from countries with very different health care traditions, and are commonly unfamiliar with the requirements of the health care system in the host country. Also, IMs tend to be unaware of what services exist and are available.

d. *Care providers’ lack of awareness of the legal entitlement of IMs to mental health care*

*“Most of the irregular migrants don’t have the knowledge of the Belgian health care system, that they have access to medical care. When they come here, they are lost and they don’t understand they have that right [to obtain a medical card].” (Belgium_19)*"

A lack of awareness among care providers of what entitlements to health care are in place for IMs was reported as a significant barrier, as it leads to IMs being turned away or left with a minimum of medical attendance despite having the right to receive care.

*“Many times, they [IMs] fail right at the entrance, in the reception: administrative staff ask for a series of documents, and require the payment of taxes, that often are not applicable to that person.” (Portugal_132)*"

In countries with limited access to care for mentally ill IMs, this lack of awareness amongst staff leads to problems with the interpretation of what constitutes emergency care and who is in need of it.

*“Hospitals are supposed to treat anyone in case of an emergency – yes, but what is an emergency?” (Austria_7)*"

Experts pointed out that decisions that should be made by clinicians are often left to administrative staff with control over access to care. This in turn can lead to IMs being denied access due to arbitrariness and sometimes discrimination.

e. *Lack of competencies and resources for treating migrants with mental health problems*

*“It depends on how willing the psychiatrist is to say what an urgent need is… We have heard that consultants are not willing to make that decision – the overseas payment officer makes the decision although the decision is supposed to be a clinical one, i.e. a clinician’s responsibility.” (UK_173)*"

It was noted that services often lack the competencies and resources for treating migrants in general. This includes a shortage of mental health professionals with knowledge and experiences that are specifically relevant for the treatment of migrants (e.g. expertise in treating traumatised people). The problem was reported for both public mental health services and NGOs. The lack of professional interpreters with training in mental health was seen as particularly problematic due to the importance of language and accurate communication for diagnosing and treating patients with mental disorders. Also, even when medical treatment is reimbursed, other types of services (e.g. interpreting service or psychotherapy) often are not.

*“Another obstacle could be language because not everyone works with interpreters. Linguistic difficulties can sometimes give the impression that the patient is psychologically disturbed when all he is trying to do is express things that belong to another culture.” (France_47)*"

Other related difficulties included the poor quality of interpreting services generally and the increased time required for communicating through an interpreter. Finally, the experts also identified insufficient cultural competencies, a shortage of multilingual staff and a lack of information material in different languages as contributing to this barrier.

f. *General shortage of resources in services providing mental health care*

*“There’s a great difficulty in finding mental health services, which are culturally sensitive and culturally competent.” (Portugal_132)*"

A general shortage of resources and limited capacities in mental health services (including mainstream services and NGOs) were reported as a barrier in a number of countries. It was noted that IMs often find themselves at the “back of a queue” for receiving care. Problems include long waiting lists; restricted availability of psychotherapy and psychological treatments; a shortage of social workers in hospitals; a lack of mental health components in primary care services, aftercare and coordination; no provision of day care; and no or difficult access to information on mental health services.

*“We are severely undersized compared to the very high demand.” (France_47)*"

Limited resources mean that NGOs frequently have to rely on volunteer staff, which sometimes affects the quality of services provided.

g. *Lack of trust in health and social care professionals and institutions*

*“XX [an NGO] works with medical staff, often volunteers, and it takes more time to do that sensitisation work because the staff is more different, the teams change more often, the knowledge of the specific problems of the illegals is to be renewed with each doctor.” (Belgium_19)*"

Experts reported that IMs frequently do not have enough trust in health and social care professionals and institutions as they fear that their personal details could be passed on to immigration authorities, potentially leading to deportation.

h. *IMs’ complex needs beyond the capacity of mental health services*

*“People who are undocumented are fearful of deportation, about even coming forward to a service, fear of being sent home …” (Ireland_82)*"

Experts emphasised the link between the complex living circumstances of IMs and their problems in obtaining care or maintaining treatment. IMs often live in unfavourable socio- economic situations, suffer from fear and insecurity regarding their immigration status, and are socially isolated. Basic living conditions are often compromised, with many IMs being homeless and without sufficient money for food, hygiene or transport. In some cases they even have to hide from authorities. In such situations survival takes priority over looking after a person’s mental health and seeking treatment.

*“The problem is the struggle to survive, to exist, to fulfil the basic needs: somewhere to sleep, a roof over the head, food, and I keep mentioning the toilet paper, thetoothpaste is not as important as the toilet paper, especially for women, one should not underestimate personal hygiene. So, only if those basic needs are fulfilled, they may be somehow able to attend to treatment for a longer period of time…” (Austria_7)*"

A lack of social welfare and housing entitlements makes it even more difficult for services to help IMs with mental health problems and to address their complex needs. Access to mental health care may be limited due to unsolved social welfare problems (e.g. being homeless).

*“Sometimes, after the admission interview, mental health care is not offered if basic personal needs, such as accommodation, are not met, since it is assumed that this undermines any therapeutic effect.” (The Netherlands_109)*"

The adequate provision of social care was seen by the experts as a necessary part of mental health treatment.

*“If the man is socially destitute at the same time, he will need support and assistance from the social services and then we could deal with his exile and migrant status….If he is experiencing delirium, he should receive the same care as any other delirious patient. If he is not delirious, we have to find out why this person is totally isolatedand provide the appropriate response on the social or psychological level…The social aspect is important: providing them with a structure, support and care.” (France_46)*"

Social deprivation was often regarded as causing or contributing to psychological problems in IMs. Consequently the needs of IMs cannot be met without more comprehensive care packages, including support for welfare problems.

*“Undocumented migrant status can bar someone’s access to care and [they] wouldn’t get holistic services – whereas lack of housing may be the main reason for the psychiatric episode.” (UK_173)*"

### Overcoming barriers

Despite this long list of barriers to mental health care for IMs identified by experts, at least some type of mental health care was provided for IMs in most countries. We identified six themes relevant to different ways of overcoming barriers to care (Table 3).

a. *Treating IMs despite legal or administrative restrictions*

**Table 3:** Overcoming barriers - frequencies of themes sorted by the number of countries from highest to

**Themes**	**14 countries 25 experts**	**Countries**
NGOs as important players in mental health care for IMs (b)	14 countries 24 experts	Austria, Belgium, Czech Republic, France, Germany, Hungary, Ireland, Italy, The Netherlands, Poland, Portugal, Spain, Sweden, United Kingdom
Treating IMs despite legal or administrative restrictions (a)	13 countries 20 experts	Austria, Belgium, Czech Republic, France, Germany, Hungary, Ireland, Italy, The Netherlands, Poland, Portugal, Sweden, United Kingdom
Referrals arising from informal networks and committed professionals (f)	13 countries 19 experts	Austria, Belgium, France, Germany, Hungary, Ireland, Italy, The Netherlands, Poland, Portugal, Spain, Sweden, United Kingdom
Treating IMs despite costs not being reimbursed (c)	10 countries 14 experts	Austria, Belgium, Czech Republic, Germany, Ireland, The Netherlands, Poland, Portugal, Sweden, United Kingdom
Helping without an official mandate (d)	9 countries 11 experts	Austria, Czech Republic, Germany, Ireland, Italy, The Netherlands, Poland, Sweden, United Kingdom
Consequences of mental health treatment for the legal status of IMs (e)	6 countries 7 experts	Austria, France, Germany, Ireland, Poland, United Kingdom

Experts reported that some services and professionals treat patients free of charge despite legal restrictions, knowing that the costs are not covered. When faced with an ethical dilemma between following legal and administrative rules that required them to turn IMs away, or providing care nonetheless, they tended to choose the later option. They would also allow for flexibility in administrative procedures, as IMs often do not comply with requirements. Such support was reported particularly for denominational hospitals and in NGOs. However, experts pointed out that staff in general hospitals, general practitioners and medical specialists also frequently turned a blind eye to statutory restrictions and treated IMs.

*“… some doctors really engage in help. Quite often the doctors use their own contacts to treat such patients or look for some ways to break the regulations and treat a patient without medical insurance.” (Poland_122)*"

What enables professionals to defy institutional rules and provide care for IMs in need? One expert reported that in his organisation there was no requirement to register patients’ personal details and consequently he was able to avoid a potential conflict within the institution:

*“And like I said, I'm able to offer counselling. So I don't look at where this person comes from. Because I work in this facility, I don't have to register everyone and do not have to refer them somewhere else. So I have this possibility. But there are places, facilities, that don't have this possibility…” (Germany_75)*"

The decision as to whether to provide care for an IM or turn him/her away was described as a matter of individual conscience, but also as a decision that is influenced by the given institutional circumstances.

b.*NGOs as important players in mental health care for IMs*

*“It's probably a matter of conscience whether an employee, doctor, psychiatrist treats such a person or not. What I would do, if I worked in a psychiatric hospital, I don't know, whether I would be in a position to care for these people and treat them." (Germany_75)*"

NGOs were considered important for overcoming barriers to mental health care for IMs. There were two reasons for this. Firstly, NGOs provide a broad range of services for this group such as interpreting, culturally sensitive services, psychotherapy and/or counselling, treatment for victims of torture, active outreach, provision of information, material support, social welfare, legal counselling and mental health advocacy.

*“We can provide psychological help in eight languages, with help from professional translators if necessary; we also have cultural mediators to help people from different backgrounds. We provide legal advisory and social workers. We also provide help in contacts with health care – hospitals, doctors, and in contacts with any official institutions.” (Poland_121)*"

A single NGO may offer only specific services (e.g. medication, crisis intervention) for a limited time, but this was already considered an improvement in the context of what is usually available for this group.

*“…she [an IM seeking mental health care] would get medication and we would offer her psychotherapeutic crisis intervention, so she has the chance to communicate, talk about her fears.” (Austria_7)*"

Secondly, NGOs also provide low threshold access to care. IMs appear to find it easier to trust staff in NGOs and are less afraid of being reported, since anonymity was better protected. The humanitarian character of NGOs plays a crucial role. According to the experts, IMs believe that NGOs will support them, even when no other services are willing to help.

c.*Treating IMs despite costs not being reimbursed*

*“We've [NGO] been working as a contact point for 13 years now, and word has gotten around that we're not endangering anybody or calling the immigration authorities, and that the people we're referring to are trustworthy. So some mutual trust hasdeveloped over the years, people who have had a good experience with us recommend us among their communities.” (Germany_66)*"

The questions of who should bear the treatment costs, and whether it is fair that services have to meet the costs for IMs themselves, were reported as a matter of significant concern. When services are able to find ways to shift certain costs within their budget, they are in a position to make mental health care available to their IM patients. However, they have to be more selective in the type of service they provide.

d.*Helping without an official mandate*

*“We give some support for medication that we can afford and we pay for some tests. However, we are not able to pay for all the tests.” (Portugal_133)*"

NGOs in general, and especially those providing services for homeless people and refugees/asylum seekers, were identified as a notable first point of contact for IMs. However, because the service profile of such NGOs usually do not include help for IMs and resources are typically limited, the decision to provide care is often challenging. Experts reported that many services treat IMs despite these restrictions.

*“They would offer necessary medical and psychosocial help, sometimes also shelter, food, clothing, etc. For asylum seekers who have exhausted all legal procedures this ismore difficult since it is illegal to offer accommodation for the night for them.” (The Netherlands_108)*"

In some cases experts noted that services could potentially threaten their own existence if it became known that they were supporting a group which they were not supposed to support.

e. *Consequences of mental health treatment for the legal status of IMs*

*“The health care for illegal immigrants is mostly provided underground or by NGOs which do it on the conditions that it is illegal according to the country’s legislation.” (Sweden_164)*"

Experts pointed out that the legal procedures involved in admission and treatment of a patient with a serious mental disorder, who is a danger to himself/herself or others, can result in the patient being discovered as an IM by authorities. Consequently, these procedures may contribute to a risk of being deported.

*“Only in extreme cases – if he is dangerous for others or himself, he would be referred for observation to hospital or treatment without his consent – he would not have to pay then but his status would be a strong issue then, it would have to be clarified. Because, if there is an admission without a consent – there must be legal procedures involved, including a judge’s opinion, a court hearing etc. So it would be difficult to avoid a question on his legal status. It is possible that after his state is stabilised, hewould be deported – it depends on the case.” (Poland_121)*"

While this may constitute a barrier, it also opens up the possibility of providing IMs with the mental health care they need, and sometimes even of an illegal status being regulated, at least for a certain period of time.

*“There are numerous possibilities in France to help people with serious medical or psychological disorders which protect them from deportation in this country, because we first have to treat them and then decide what to do.” (France_46)*"

Providing legal support and advice for IMs was mentioned by experts as an important complementary aid, especially as some of them may be successful in legalising their status and thus obtaining better access to mental health care.

f. *Referrals arising from informal networks and committed professionals*

*“…if the hospital's social workers are very experienced in this area, they might know that coverage could be arranged by securing his immigration status.” (Germany_66)*"

Experts emphasised that, commonly, IMs do not directly approach services that are in a position to care for their mental health needs. They have a tendency to contact organisations they trust, regardless of their specific expertise. Many NGOs are highly specialised, addressing specific issues and operating with restricted resources, which limits the range of services they can offer:

*“A parallel network of non-governmental organisations is being set up to attend them, but they cannot address the needs of all these persons.” (Spain_149)*"

Thus, when further referral is needed, staff often select the appropriate service and coordinate referrals and interventions. The information on referral options is usually based on personal knowledge and experience, as well as on the efforts of professionals to maintain good relationships with staff from other services. However, it is not only NGOs that rely on their informal networks. Staff in mainstream services also use such networks for finding appropriate care for IMs with mental health problems, which can be specialised mental health services, social services or both, depending on the specific needs of a patient.

*“They have contacts and know where to refer such person, to which trusted doctor or clinic. They would refer such a person on the basis of their personal contacts.” (Poland_121)*"

## Discussion

### Main findings

This qualitative study of experts’ views about mental health care for IMs in the capital cities of 14 European countries identified barriers to mental health care, and explored ways of how these barriers are overcome in practice, when networks of supportive professionals and services seek to provide care for this group despite these barriers.

In countries with no legal access to health care for IMs, this lack of protection was identified by the experts as the main barrier for IMs in need of treatment. However, even in countries where obtaining health care is legally possible, other barriers arise that often prevent actual access. When we applied a classification proposed by Karl-Trummer et al. [19], whereby the participating countries were divided into those with no legal entitlement to health care at all (except emergency care) and those with partial or full legal access, most of the barriers reported were still relevant in both groups. These other barriers included a lack of awareness of legal entitlements of IMs (or access to emergency care) by both IMs and providers, lack of specific competencies and resources in mental health care providers for treating migrants in a culturally sensitive way - which includes providers responding to and understanding of culturally influenced needs, expressions, believes and behaviours - a general shortage of resources in mental health services, IMs’ distrust of health and social care professionals, and the complex needs of IMs which often cannot be met within the capacities of mental health services.

Despite these barriers, some mental health care is actually provided to IMs even in countries where IMs have no legal entitlement. Experts pointed out that although there are ways to overcome some barriers, professionals in services face various difficulties and dilemmas in providing care. If services and staff decide to treat IMs despite institutional restrictions, they are left with a number of practical problems such as the fact that the costs may not be covered. Also, services may jeopardise their own existence if it becomes known that they are helping a group that they are not supposed to help. This especially applies to services caring for homeless and asylum seekers/refugees, which often act as a contact point for IMs. Denominational hospitals and some NGOs may have no or fewer restrictions in their access rules and a clear humanitarian mission of helping people in need. They may therefore find it easier to treat IMs.

Most of the identified ways of overcoming barriers are applicable to health care in general, but some are specific to mental health care. IMs receiving treatment may be reported to authorities and consequently get deported, however, it is also possible that in some countries a mental health diagnosis can protect an irregular migrant from deportation or is even helpful for legalising his/her status.

### Strengths and limitations

The study has a number of strengths, such as a carefully designed methodology and an international perspective. Although experts with knowledge and experience in the field of mental health care for IMs were difficult to identify, a total of 25 experts from 14 European countries were interviewed and provided insights into this field of mental health care.

The recruitment of these experts, however, may be subject to bias. Since experts were selected based on the local knowledge and experience of the research teams, the recruitment was opportunistic and may have been inconsistent. The three countries where only one expert was identified provided either no legal access (Austria and Sweden) or only limited access (UK) to health care for IMs, which reduced the chances to find experts familiar with the actual provision of care. A further possible limitation of the study is that some interviewees might have had personal or political agendas regarding mental health service provision for IMs, and their views may reflect their specific subjective experiences. Not all interviewed experts were mental health professionals; some of them had a professional background in general health care or social care. However, all of them had experience in facilitating access to mental health care for IMs. The experts’ views were restricted to mental health care in European capital cities and findings may not necessarily apply to different settings and geographical locations.

The views of IMs themselves were not obtained. Additional barriers faced by IMs might therefore remain unreported. Yet, on most issues we found a wide consensus of experts across Europe, which may indicate the validity of the findings despite certain methodological shortcomings of the study.

### Comparison with the literature

#### Barriers to mental health care

The current study supports the previous finding by Ruiz-Casares et al.’s [25] that although legal frameworks may be helpful in identifying differences between countries with respect to entitlements to health care, they say little about the extent to which IMs can gain access to health services in practice . Furthermore, once legal barriers to access health care are removed, other issues arise [26]. Other than legal and administrative barriers, we did not find substantial differences in the frequencies of barriers reported between the countries with different legal entitlement to health care.

Concerning the administrative barriers identified in our study, similar findings were reported by a study examining general health care utilisation by female IMs in the Netherlands [12]. Administrative staff’s lack of knowledge of health care entitlements was also identified as a barrier by Médecins du Monde in their European Observatory on Access to Healthcare Report [10]. Having an IM status implies that, even if IMs have the right to health care, they may not be in a position to claim these rights. The World Health Organisation (WHO) states in its Factsheet no. 31 “Right to Health” that, if health care is restricted to “essential care” or “emergency health care”, laws and practices may be discriminatory [27]. Since a “mental health emergency” is not well defined, decisions on what constitutes an emergency are not always based on objective criteria. However, Karl-Trummer et al. [19] pointed out that vague access rules may be interpretable in favour of the patient; that is, by formulating a case as an emergency.

As experts reported in the present study, finding culturally sensitive care can be challenging. Migrants may have specific health needs, particularly mental health needs, which are not always appropriately attended to in mainstream services. This has been pointed out in many other studies that pertain to health care for migrants generally [28-32]. Recognition, diagnosis and treatment of mental disorders and psychosocial problems are highly dependent on the language skills and the cultural competencies of health care professionals. Such skills have previously been noted as being of particular importance when addressing migrants’ mental health [33].

Health care systems in Europe vary in their quality and the resources available. If the lack of resources in mental health care affects the general population of a country, it is likely to affect those with irregular status even more as the European Union Agency for Fundamental Rights [6] has recently noted.

Finally, IMs’ difficult life circumstances (e.g. poor socio-economic conditions) have been reported as barriers to accessing and receiving health care in many studies [10,12-14]. The experts in our study outlined that, without finding appropriate solutions for IMs’ unmet social needs, the treatment of specific mental health disorders may be impossible.

#### Overcoming barriers

The findings show that, despite the various barriers, IMs do get some help for mental health problems in most countries. Informal networks of supportive professionals and services providing care beyond their remit exist in practically all of the countries in this study. These networks give higher priority to human rights than to legal regulations and are creative in finding solutions in order to provide care for IMs with mental health problems. This finding is supported by reports on health care provision for IMs in Austria and Germany, where services catering for IMs rely on a network of medical doctors who are willing to accept their referrals and treat patients free of charge [34-36]. A survey on the treatment of IMs in Germany found that every second physician reported to have had some experience with treating IMs [37].

Where the public health care system fails to provide mental health care for IMs because of legal restrictions or lack of adequate resources (e.g. interpreting services), NGOs frequently attempt to fill that gap. This important role of NGOs, emphasised by experts in the present study, is consistent with the literature on general health care for IMs [19,38,39] and previous reports on mental health care for this group [6]. However, NGOs usually have fewer facilities than mainstream services and the quality of care might sometimes be compromised due to limited resources, as Biswas et al. [40] also noted in their study on IMs in Denmark.

The support for IMs in mainstream mental health services depends on the good will of staff, but also on institutional arrangements, e.g. issues such as whether costs can be shifted within a budget, whether unpaid bills are accepted by the administration, and whether there is a possibility of treating a patient without reporting it [41,42]. As Jensen et al. [43] pointed out, most general practitioners in their study treated IMs despite limited legal entitlement, but they had to be creative when further diagnostic procedures were required (e.g. using personal contacts or sending in test samples in their own name).

The present study shows that the lack of legal entitlements to care and consequent need for informal supportive networks can create many dilemmas for professionals and services providing mental health care to IMs. Firstly, providing care without an official mandate and in spite of restricted resources means that services are jeopardising their funding and resources for treating their “regular” patients. Secondly, they are faced with uncovered costs and difficult procedures of reimbursement. When the economic survival of services is at stake, the support for IMs can decrease, and IMs that otherwise would be treated may be turned away. In such cases humanitarian behaviour becomes synonymous with loss of income [11]. The tension between the two reference systems governing health care and their two sets of rules, *i.e.* the public administrative system on one hand and the humanitarian principle on the other [44], becomes evident in access to health care for IMs [45]. Jensen et al. [43] found that the lack of legal entitlements to health care for IMs resulted in substantial concerns among health care professionals about how to handle encounters with this group. For example, professionals expressed uncertainty about whether to prescribe medicine or demand payment for treatment first. This was often further exacerbated by the absence of organisational policies and guidelines on how to deal with IMs [40,41]. Ethical considerations became especially important in countries where treating IMs’ mental health problems could contribute to the risk of them being reported to authorities and removed from the country.

### Implications for improving practice

The results of the present study have implications for improving practice. Firstly, from a humanitarian point of view, access to mental health care for IMs should be ensured at the level of policies and legislation. As the lack of awareness about legal entitlements was one of the main barriers identified in this study, the relevant information should be clearly communicated by care providers and professional associations to all health care professionals and administrative staff. Migrant communities and associated organisations should ensure that the same information about entitlements to health care reaches IMs. Furthermore, administrative arrangements should be adapted to accommodate the frequent inability of IMs to prove identity or residency. Cooperation between NGOs and mainstream mental health services is essential to ensure that IMs receive appropriate care. Mental health services should be supported in providing culturally sensitive care, including the provision of interpreting services and/or multilingual staff. As the complex life circumstances of IMs tend to interfere with both access to and maintenance of mental health treatment, the provision of relevant legal advice and social counselling may help IMs to obtain appropriate mental health care over longer periods of time if required.

## Conclusions

IMs endure significant hardship and can be exposed to a number of risk factors for developing mental health problems. The broad range of barriers to mental health care they encounter is to some extent overcome by a network of supportive professionals and organisations who seek to provide care for this group in spite of restrictions. However, the resources of these networks are limited. Health care professionals who treat irregular migrants face an ethical dilemma in cases where providing care means bypassing legal regulations, or when treatment may have an effect on patients’ legal status and their stay in the country. The findings of this study might support legislative changes for ensuring that access to appropriate mental health care is guaranteed. However, further efforts to communicate information about entitlements to both service providers and IMs are also required. Providing sufficient resources to care providers to implement culturally sensitive mental health care is also essential. As IMs’ difficult life circumstances often represent a barrier to receiving effective treatment, appropriate social care support needs to be provided.

## Competing interests

The authors declare that they have no conflict of interest.

## Authors’ contributions

CS, AM, SP, HB, RC, JMDO, ED, AG, TG, PH, UK, MW, PN, AHS, JJFS and HK all made a substantial contribution to the design of the interview study, data collection, interpretation of findings and critical revision of drafts. CS, AM, SP and HK contributed to the analysis of findings and drafting of the manuscript. All authors read and approved the final manuscript.

## Pre-publication history

The pre-publication history for this paper can be accessed here:

http://www.biomedcentral.com/1471-2458/12/367/prepub

## References

[B1] Council of Europe: Recommendation Rec 18 of the Committee of Ministers to member states on health services in a multicultural society 2006 Strasbourg

[B2] Council of Europe Bratislava Declaration on health, human rights and migration 2007 Bratislava

[B3] European Parliament European Parliament resolution of 8 March 2011 on reducing health inequalities in the EU (2010/2089(INI)) 2011 Strasbourg [http://www.europarl.europa.eu/sides/getDoc.do?type=REPORT&reference=A7-2011-0032&language=EN]

[B4] Jandl M Vogel D Iglicka K Clandestino - Undocumented Migration: Countingthe Uncountable. Data and Trends Across Europe. Report on methodological issues [http://clandestino.eliamep.gr/wp- content/uploads/2009/10/clandestino_report-on-methodological-issues_final12.pdf]

[B5] PICUM – Platform for International Cooperation on Undocumented Migrants PICUM’s Main Concerns about the Fundamental Rights of Undocumented Migrants in Europe 2009 Brussels

[B6] FRA – European Union Agency for Fundamental Rights Migrants in an irregular situation: access to healthcare in 10 European Union Member States 2011 Luxembourg

[B7] EUGATE Best Practice in Health Services for Immigrants in Europe[http://www.eugate.org.uk/]

[B8] PICUM – Platform for International Cooperation on Undocumented Migrants Undocumented Migrants’ Health Needs and Strategies to Access Health Care in 17 EU Countries. Summary report in the framework of the project Healthcare in NowHereland 2010 Brussels

[B9] HUMA network Access to health care for undocumented migrants and asylum seekers in 10 EU Countries 2009 Paris: Law and Practice

[B10] Médecins Du Monde - European Observatory on Access to Healthcare Access to healthcare for undocumented migrants in 11 European countries 2009 2008 Survey report

[B11] Romero-Ortũo R Access to health care for illegal immigrants in the EU: should we be concerned? European Journal of Health Law 2004 11 245 272 10.1163/1571809042388572

[B12] Schoevers MA Loeffen MJ Muijsenbergh ME Lagro-Janssen ALM Health care utilisation and problems in accessing health care of female undocumented immigrants in the Netherlands International Journal of Public Health 2010 55 421 428 10.1007/s00038-010-0151-6 20502936 PMC2941053

[B13] Castañeda H Illegality as risk factor: A survey of unauthorized migrant patients in a Berlin clinic Social Science & Medicine 2009 68 1552 1560 10.1016/j.socscimed.2009.01.024 19246143

[B14] Wolff H Epiney M Lourenco AP Costanza MC Delieutraz-Marchand J Andreoli N Dubuisson JB Gaspoz JM Irion O Undocumented migrants lack access to pregnancy care and prevention BMC Public Health 2008 8 93 10.1186/1471-2458-8-93 18366690 PMC2323378

[B15] Lindert J Schouler-Ocak M Heinz A Priebe S Mental health, health care utilisation of migrants in Europe European Psychiatry 2008 23 14 20 18371575 10.1016/S0924-9338(08)70057-9

[B16] Moussaouri D Agoub M Bhugra D, Gupta S Migration and Mental Health Risk and protective factors in mental health among migrants 2011 Cambridge: Cambridge University Press 98 106

[B17] European Migration Network Illegally Resident Third Country Nationals in EU member States 2007 Geneva: state approaches towards them, their profile and social situation

[B18] Stanciole AE Huber M Access to Health Care for Migrants 2009 Vienna: Ethnic Minorities and Asylum Seekers in Europe

[B19] Karl-Trummer U Novak-Zezula S Metzler B Access to health care for undocumented migrants in the EU: A first landscape of NowHereland Eurohealth 2010 16 13 16

[B20] Hsieh HF Shannon SE Three Approaches to Qualitative Content Analysis Qualitative Health Research 2005 15 1277 1288 10.1177/1049732305276687 16204405

[B21] Pope C Ziebland S Mays N Pope C, Mays N Qualitative research in health care Analysing qualitative data 2006 3 rd Blackwell Publishing 63 81 10.1136/bmj.320.7227.114 PMC1117368 10625273

[B22] Patton MQ Qualitative Research and Evaluation Methods 2002 3 Thousand Oaks, CA: Sage

[B23] Silverman D Interpreting Qualitative Data: Methods for Analysing Talk, Text and Interaction 2001 2 London: Sage

[B24] Morgan DL Qualitative content analysis: a guide to paths not taken Qualitative Health Research 1993 3 112 121 10.1177/104973239300300107 8457790

[B25] Ruiz-Casares M Rousseau C Derluyn I Watters C Crépeau F Right and access to healthcare for undocumented children. Addressing the gap between international conventions and disparate implementations in North America and Europe Social Science & Medicine 2010 70 329 336 10.1016/j.socscimed.2009.10.013 19892452

[B26] Torres-Cantero AM Miguel AG Gallardo C Ippolito S Health care provision for illegal migrants: may health policy make a difference? European Journal of Public Health 2007 17 483 485 17208953 10.1093/eurpub/ckl266

[B27] UNCHR The Right to Health. Factsheet No. 31 2008 OHCHR-WHO

[B28] Carta MG Bernal M Hardoy MC Haro-Abad JM the “Report on the Mental Health in Europe” working group Migration and mental health in Europe (the state of the mental health in Europe working group: appendix I) Review Clinical Practice and Epidemiology in Mental Health 2005 I 13 16135246 10.1186/1745-0179-1-13 PMC1236945

[B29] Mladovsky P Research Note produced for the European Commission as part of the Health and Living Conditions Network of the European Observatory on the Social Situation and Demography Migration and health in the EU

[B30] Padilla B Portugal R Ingleby D De Freitas C Lebas J Fernandes A, Pereira Miguel J Health and Migration in the European Union: Better Health for All in an Inclusive Society Health and Migration in the European Union: Good Practices 2009 Lisbon 101 115

[B31] Priebe S Sandhu S Dias S Gaddini A Greacen T Ioannidis E Kluge U Krasnik A Lamkaddem M Lorant V Puigpinósi Riera R Savary A Soares JJF Stankunas M Straßmayr C Wahlbeck K Welbel M Bogic M EUGATE group Good practice in health care for migrants: views and experiences of care professionals in 16 European countries BMC Public Health 2011 11 187 10.1186/1471-2458-11-187 21439059 PMC3071322

[B32] Devillé W Greacen T Bogic, Dauvrin M, Dias S, Gaddini A, Koitzsch Jensen N, Karamanidou C, Kluge U, Mertaniemi R, Puigpinós i Riera R, Sárváry A, Soares JJF, Stankunas M, Straßmayr C, Welbel M, Priebe S: Health care for immigrants in Europe: is there still consensus among country experts about principles of good practice?A Delphi study BMC Public Health 2011 11 699 10.1186/1471-2458-11-699 21914194 PMC3182934

[B33] Tribe R Bhugra D, Gupta S Migration and Mental Health Migrants and mental health: working across culture and languages 2011 Cambridge: Cambridge University Press 261 273

[B34] Holzer I Rásky É Gesundheit hat Bleiberecht Das Ambulatorium Caritas Marienambulanz und seine Spezialambulanzen 2009 Vienna: Facultas 238 242

[B35] Haentjes-Bögers A Rásky É Gesundheit hat Bleiberecht Die Malteser Migranten Medizin (MMM) – Hilfe für Menschen ohne Krankenversicherung 2009 Vienna: Facultas 339 348

[B36] Hilbert T Sieveking K Hegemann T, Salman R Handbuch Transkulturelle Psychiatrie Gesundheitliche Versorgung von Menschen “ohne Papiere” 2010 Bonn: Psychiatrie-Verlag GmbH 317 329

[B37] Wiesner A Schmidt S Bergmeyer V 2008 Bruckermann U: Gesundheitsversorgung von papierlosen Menschen in Bremen. Ergebnisse einer Umfrage bei Arztpraxen im Land Bremen. Bremen

[B38] Huber M Stanciole A Wahlbeck K Tamsma N Torres F Jelfs E 2008 Bremner J: Quality in and Equality of Access to Health Care Services. Vienna

[B39] Waller H Gesundheitsprobleme und Gesundheitsversorgung von Menschen in der aufenthaltsrechtlichen Illegalität: Deutschland und Italien im Vergleich Gesundheitswesen 2008 70 4 8 10.1055/s-2007-1022527 18273758

[B40] Biswas D Kristiansen M Krasnik A Norredam M Access to healthcare and alternative health-seeking strategies among undocumented migrants in Denmark BMC Public Health 2011 11 560 10.1186/1471-2458-11-560 21752296 PMC3163547

[B41] Priebe S Bogic M Adany R Bjerre NV Dauvrin M Deville W Dias S Gaddini A Greacen T Kluge U Ioannidis E Jensen NK Puigpinós I Riera R Soares JJF Stankunas M Straßmayr C Wahlbeck K Welbel M McCabe R for the EUGATE study group Migration and Health in the European Union Good practice in emergency care: views from practitioners 2011 First Maidenhead: Open University Press 213 226

[B42] Dauvrin M Lorant V Sandhu S Devillé W Dia H Dias S Gaddini A Ioannidi E Kluge U Koitzsch Jensen N Mertaniemi R Puigpinós i Riera R Sarvary A Straßmayr C Stankunas M Soares J Welbel M Priebe S Healthcare for irregular migrants: pragmatism across Europe. A qualitative study BMC Research Notes 2012 5 99 10.1186/1756-0500-5-99 22340424 PMC3315408

[B43] Jensen NK Norredam M Draebel T Bogic M Priebe S Krasnik A: Providing Medical Care for Undocumented Migrants in Denmark: What Are the Challenges for Health Professionals? BMC Health Services Research 2011 11 154 10.1186/1472-6963-11-154 21711562 PMC3150245

[B44] Düvell F Book of Solidarity 1 Who else if not us?” Ethics in Immigration and Social Work with undocumented Migrants 2002 Antwerp: De Wrikke 84 90

[B45] Scott P Undocumented Migrants In Germany And Britain: The Human “Rights” And “Wrongs” Regarding Access To health Care[http://www.sociology.org/content/2004/tier2/scott.html]

